# Proteins That Promote Filopodia Stability, but Not Number, Lead to More Axonal-Dendritic Contacts

**DOI:** 10.1371/journal.pone.0016998

**Published:** 2011-03-07

**Authors:** Pamela Arstikaitis, Catherine Gauthier-Campbell, Kun Huang, Alaa El-Husseini, Timothy H. Murphy

**Affiliations:** Department of Psychiatry and The Brain Research Centre, University of British Columbia, Vancouver, British Columbia, Canada; Université de Technologie de Compiègne, France

## Abstract

Dendritic filopodia are dynamic protrusions that are thought to play an active role in synaptogenesis and serve as precursors to spine synapses. However, this hypothesis is largely based on a temporal correlation between filopodia formation and synaptogenesis. We investigated the role of filopodia in synapse formation by contrasting the roles of molecules that affect filopodia elaboration and motility, versus those that impact synapse induction and maturation. We used a filopodia inducing motif that is found in GAP-43, as a molecular tool, and found this palmitoylated motif enhanced filopodia number and motility, but reduced the probability of forming a stable axon-dendrite contact. Conversely, expression of neuroligin-1 (NLG-1), a synapse inducing cell adhesion molecule, resulted in a decrease in filopodia motility, but an increase in the number of stable axonal contacts. Moreover, RNAi knockdown of NLG-1 reduced the number of presynaptic contacts formed. Postsynaptic scaffolding proteins such as Shank1b, a protein that induces the maturation of spine synapses, increased the rate at which filopodia transformed into spines by stabilization of the initial contact with axons. Taken together, these results suggest that increased filopodia stability and not density, may be the rate-limiting step for synapse formation.

## Introduction

In the CNS, synapse formation between axons and dendrites is a regulated process involving the coordinated actions between presynaptic axons and postsynaptic dendrites [1]. Coordination of this physical interaction between pre- and postsynaptic cells is thought to occur via dendritic filopodia that contact and recruit passing axons [2], [3], [4]. Dendritic filopodia are thin, headless protrusions ranging from 2–25 µm in length that are filled with bundles of actin and extend from the cell surface [5], [6], [7]. Early in development, immature neurons are littered with highly motile dendritic filopodia. As the brain matures, these abundant and motile filopodia are replaced with more stable spine synapses [8].

Multiple studies suggest that after filopodia participate in synaptic contact formation, they transform to more stable dendritic spines through the actions of synapse-inducing factors [9], [10], [11], [12] and neuronal activity [13], [14], [15]. However, whether the increased density and motility of filopodia are associated with the formation of dendritic spine synapses is controversial. One previous imaging study showed highly motile filopodia mainly form transient interactions with presynaptic terminals [16]. Another study revealed that neuronal membrane glycoprotein M6a-induced filopodia are highly motile and become stabilized upon contact with presynaptic regions [17]. In contrast, a recent study found that a reduction in the motility of EphB-induced filopodia led to a decreased rate of synaptogenesis [18].

To date, it is unclear how different molecules behave to initiate synaptic contact formation and transform filopodia to spines. We address this by comparing the effect that specific molecules, known to play a role in synapse formation, have on filopodia dynamics. Shank1b and NLG-1 proteins are two major components of the postsynaptic density (PSD) and influence the maturation of synapses. Shank1b promotes maturation of dendritic spines [19], while its dominant negative mutant causes a reduction in spine size and density [20]. NLG-1, a synaptic cell adhesion molecule, initiates communication between pre- and postsynaptic sites and influences the development of functional synaptic terminals [21]. We recently showed Cdc42 (CA)-Palm has potent affects on inducing dendritic spines in mature neurons [22], however its role in filopodia dynamics and synapse formation remain less clear. Here, we will investigate the origin of dendritic spines induced by Cdc42 (CA)-Pam, NLG-1 and Shank1b by examining how these proteins impact the motility of dendritic filopodia and their role in forming stable axo-dendritic contacts.

Previously we identified the palmitoylated protein, GAP-43, as a potent inducer of filopodia [5], [23]. We now use the filopodia-inducing motif of GAP-43 (GAP 1–14) as a tool to examine how increasing the presence of motile filopodia affects synapse formation. It should be noted that the full length GAP-43 protein localizes to presynaptic growth cones *in vivo*. Thus, we use this filopodia-inducing motif to strictly manipulate dendritic filopodia number and motility, but its presynaptic role is not addressed in this study. It is possible that molecules such as GAP 1–14 may hinder the formation of synapses by inducing highly motile filopodia that continuously sample the environment, yet require the recruitment of scaffolding proteins to form stable axo-dendritic contacts. Interestingly, the combination of a known filopodia inducing molecule, paralemmin-1, with the spine-stabilizing molecule Shank1b, results in an increase in the number of dendritic spines compared to expression of GFP or paralemmin-1 alone [5]. This suggests a role for molecules such as Shank1b and NLG-1 in the formation of stable filopodia-like protrusions that promote dendritic spines and synapse formation. Hence, enhancing the formation of filopodia may not necessarily lead to more stable axo-dendritic contacts. Rather, the production of stable synapses is dependent on key members of the postsynaptic scaffolding complex. In this study, we will examine molecules that affect filopodia elaboration and motility, versus those that impact synapse induction and maturation to better define the role of filopodia in synapse formation.

## Results

### Induction of Dendritic Filopodia by Expression of Specific Protein Motifs

**Figure 1:**
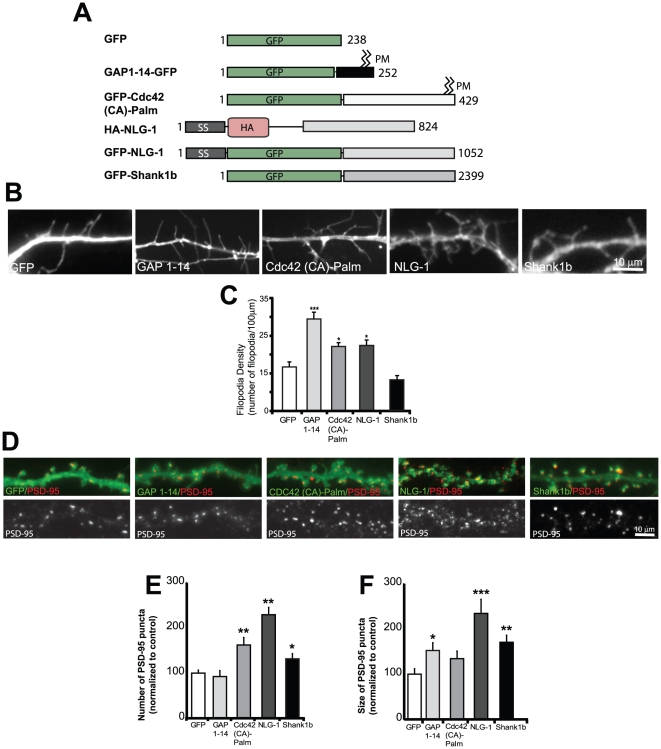
Specific synapse-inducing proteins are important for filopodia induction. (A) Schematic of the various fluorescently tagged constructs used in this study. SS-signal sequence, GFP-green fluorescent protein, HA- hemagglutinin (B) Representative images demonstrating filopodia induction by GAP 1–14, Cdc42 (CA)-Palm, NLG-1 and Shank1b. Neurons were transfected at DIV 6–7 and stained at DIV 8–9. (C) Quantification of the number of filopodia/100 µm shows that expression of GAP 1–14, Cdc42 (CA)-Palm and NLG-1 significantly increases filopodia number. In contrast, Shank1b failed to increase filopodia number. (D) Representative dendrites from neurons expressing GFP, GAP 1–14, Cdc42 (CA)-Palm, NLG-1 and Shank1b. (E) Quantification of the number of PSD-95 puncta expressed as a percentage that is normalized to control cells. Neurons expressing Cdc42 (CA)-Palm, NLG-1 and Shank1b showed an increase in the number of spines containing PSD95 puncta. In contrast, neurons expressing GAP 1–14 did not lead to an increase in the number of PSD-95 positive spines. (F) Quantification of PSD-95 puncta size. Neurons expressing NLG-1 and Shank1b showed an increase in the size of spines containing PSD95 puncta. In contrast, neurons expressing Cdc42 (CA)-Palm and GAP 1–14 showed no increase or a moderate increase in the size of PSD-95 puncta, respectively. 8–15 cells were analyzed for each group and were collected from 3 independent experiments. *p<0.05, **p<0.01, ***p<0.001. Data represent mean ±SEM. Scale bars, 10 µm.

Since filopodia have been documented to play a role in synapse formation and the transformation to dendritic spines [2], [9], [11] we compared the ability of the palmitoylated proteins GAP 1–14, Cdc42 (CA)-Palm tagged with GFP as well as the scaffolding molecules, NLG-1 and Shank1b to induce the formation of filopodia (Figure 1A). Recently, we identified the brain-specific isoform Cdc42 (CA)-Palm, which plays an important role in the formation of dendritic spines [22]. We therefore decided to compare the differential effects of these molecules in the induction of dendritic filopodia.

We first expressed these fluorescently tagged proteins (Figure 1A) to assess whether they modulate filopodia formation. Neurons at days in vitro 8–9 (DIV 8–9) expressing the palmitoylated motif GAP 1–14 or Cdc42 (CA)-Palm showed an increase in filopodia number (Figure 1B and C). Similarly, expression of NLG-1 significantly increases filopodia number (Figure 1B and C). Consistent with previous results [5], we find that Shank1b failed to enhance the density of filopodia in hippocampal neuronal cells compared to control cells, suggesting that Shank1b differentially effects the formation of filopodia compared to GAP 1–14, Cdc42 (CA)-Palm and NLG-1.

Many imaging studies provide evidence that filopodia become stabilized in more mature neurons [2], [8], [15], [24]. Here, we wanted to determine if filopodia participate as precursors and transform into dendritic spines in mature neurons. To address this issue, we overexpressed these fluorescently tagged molecules (Figure 1A) to determine whether they could alter the development of spine synapses. The presence of spine synapses was monitored by measuring the density and size of clustered endogenous PSD-95, a major scaffolding protein found at mature excitatory synapses [25]. Neurons expressing GAP 1–14, showed no change in the number of PSD-95 clusters (84.0%±11.8%) compared to control, whereas NLG-1 showed a 208.5%±14.8% increase in the density of spine synapses formed (Figure 1D, E). Therefore, high numbers of filopodia may not be sufficient to promote dendritic spine formation. Important to note is that filopodia-inducing motifs may be unable to recruit postsynaptic proteins necessary for spine stimulated formation, possibly explaining the lack of enhanced spine numbers in their presence. Furthermore, Shank1b failed to enhance filopodia density, but significantly increased the number of spines and size of PSD-95 puncta. Neurons expressing Cdc42 (CA)-Palm, on the other hand, showed a significant increase in both filopodia number (Figure 1B, C) and PSD-95 puncta density (Figure 1D, E). To summarize, proteins that efficiently increase filopodia number, such as GAP 1–14, do not necessarily lead to more spine synapses. Conversely, proteins such as Shank1b that alter synapse formation are not necessarily the most affective at inducing filopodia. These results suggest that filopodia production is not the rate-limiting step for controlling the number of spines.

If increased filopodia density does not translate into more synapses then what is the crucial step that modulates synapse formation? We next set out to determine whether filopodia serve as precursors to spines by performing timelapse imaging of neurons expressing GFP over 3 days (DIV 10–12; 24 h time points). These cells were then retrospectively labeled for GluR1 to identify mature spine synapses (Figure S1). During this period, a large number of filopodia formed and disappeared per day (33%±6.5% and 46.3%±7.8%, respectively), when neurons were examined once every 24 hours. It is conceivable that these percentages are an underestimate since only three time points were used to preserve the health of the neurons. At the same time, as filopodia appeared and disappeared, spine density increased by 10.2%±3.1% per day. Imaging analysis of GFP transfected cells revealed that 18 new spines formed during the imaging period. Only 5 of the spines appeared at sites where filopodia were present 24 h earlier, out of 306 filopodia analyzed (67 of those remain visible for 3 days). This indicates that only 3.1%±0.3% of filopodia visible at a given time point will transform into a spine within 24 h. These results reveal that a small fraction of existing filopodia transform into spines, and that ∼30% (29.2%±2.9%) of new spines appear at sites that contained filopodia at least 24 h earlier (Figure S1B). It is important to note that these results are only correlative and based on analysis of time points 24 h apart; one cannot exclude the possibility that the majority of dendritic spines emerge from transient filopodia that were not visible during the imaging period or directly emerge from the dendritic shaft.

### Dendritic filopodia use an exploratory role to form contacts with neighboring axons

During synaptogenesis, dendritic filopodia are constantly protruding and retracting in search of the appropriate presynaptic partners [2], [26]. These filopodia can engage in synaptic contacts and undergo maturation into dendritic spines [15], [27], [28], [29]. However, it is unclear whether the rate of contact initiation and stabilization between neurons can be altered by manipulating filopodia. In order to assess what proportion of filopodia form stable contacts with nearby axons, timelapse imaging was performed in cultured hippocampal neurons. A double transfection system was used in order to visualize in real time the formation of contacts between axons of DsRed-labeled neurons and dendritic filopodia from neurons expressing one of the GFP-tagged proteins, as described in Figure 1A. Cells were retrospectively immunolabeled for MAP-2, to distinguish axons from dendrites (data not shown).

Contacts between dendritic filopodia and axons that were established and subsequently lost within 1 h were classified as ‘transient’, while contacts present for the 1 h period were considered stable [2] (Movie S1). Timelapse imaging of GFP transfected cells revealed that dendritic filopodia continually interact with axons, potentially, to establish a contact with a presynaptic partner (Figure S2, Movie S2). We found that 27.9%±3.9% of existing filopodia that formed contacts with axons were transient, whereas 21.4%±4.7% were stable for at least 1 h (Figure S2A and B, Movie S3). Furthermore, 3.3%±0.9% of emerging filopodia initiate new contacts with axons (Figure S2B, Movie S2). These results reveal that filopodia are important not only for probing the environment, but also for establishing the initial contacts between neurons. It is worth mentioning that this analysis was performed on contacts between filopodia and axons en passant. In rare occasions we also observed the initiation of contact formation by axonal growth cones, however, because very few of these events were observed, the significance of this association could not be assessed (Movie S4).

**Figure 2:**
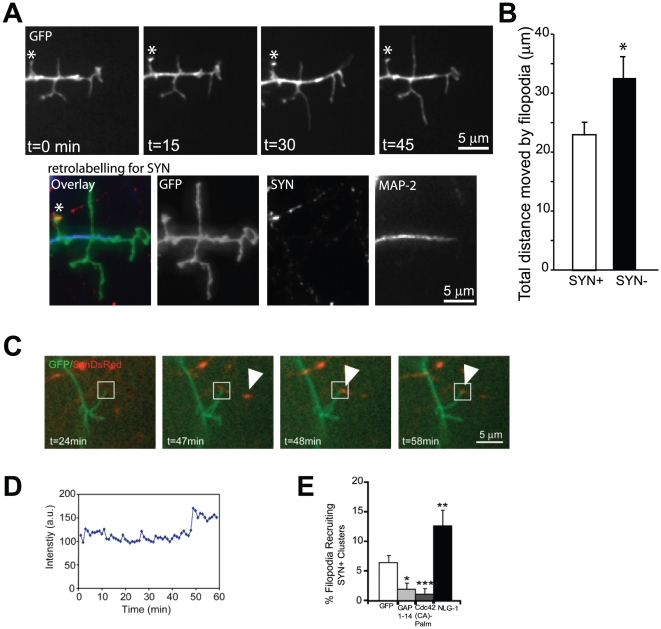
Filopodia stability and its relationship to the recruitment of presynaptic elements. (A) Example of a dendrite showing 1 stable and 3 motile protrusions. Retrolabelling for synaptophysin performed at the end of each experiment revealed that stable filopodia (labeled with *) are associated with a presynaptic terminal, positive for synaptophysin (SYN). (B) Comparison of total distance travelled by a filopodium that is associated with or without SYN. 5 filopodia were counted per cell and 8 cells were calculated from 4 independent experiments. (C) Representative timelapse images of neurons expressing GFP and Synaptophysin-DsRed. The box illustrates a filopodium (GFP) in contact with a synaptic cluster of Synaptophysin-DsRed that accumulates in brightness (shown in D) with time. (D) Intensity graph showing the increased intensity of a synaptophysin cluster with time (min). (E) Quantification comparing percentage of filopodia recruiting SYN among neurons expressing GAP 1–14, Cdc42 (CA)-Palm and NLG-1. Neurons expressing NLG-1 showed a marked increase in the percentage of filopodia that recruit presynaptic clusters compared to control neurons expressing GFP. In contrast, filopodia induced by GAP 1–14 and Cdc42 (CA)-Palm recruit significantly less SYN compared to a GFP control. *p<0.05, **p<0.01, ***p<0.001 Data represent mean ±SEM. Scale bars, 5 µm.

The transformation of filopodia to spines was preceded by a decrease in filopodial motility, an increase in the size of the tip of the filopodium to yield a spine-like protrusion [10]. Thus, the more motile the filopodium the less likely it will form a stable contact and undergo transformation to a spine. To determine if there was a correlation between filopodia motility and contact of dendritic filopodia with presynaptic clusters of synaptophysin, we performed timelapse imaging of neurons expressing GFP and performed retrospective immunolabeling to stain for endogenous synaptophysin. We found that dendritic filopodia that moved greater distances were less likely to contain a cluster of synaptophysin (filopodia that lacked synaptophysin clusters, moved 31.5±4.0 µm compared to filopodia that contained synaptophysin clusters 22.1±2.7 µm) suggesting that there is a negative correlation between the motility of a filopodium and the likelihood it will be associated with a cluster of synaptophysin (Figure 2A and B).

The ability to observe filopodia in contact with axons during live cell imaging allowed us to follow their fate over time. 6.6%±1.3% of GFP-positive filopodia stably associated with axons, but lacked presynaptic protein clusters, were found to recruit the presynaptic marker synaptophysin-DsRed within 1 h (Figure 2C,D,E). Expression of protein constructs such as GAP 1–14, and Cdc42 (CA)-Palm that result in unstable filopodia were significantly less likely to recruit synaptophysin-DsRed at sites of contact (2.2%±1.5% and 1.2%±1.1% of contacts showing recruitment). In contrast, for NLG-1 expressing cells, 11.5%±3.3% of contacts showed recruitment of synaptophysin-DsRed over the same time period (Figure 2C,D,E). These findings provide further evidence that enhanced contact stability modulated by proteins such as NLG-1 potentiate the recruitment of presynaptic elements to sites of contact between dendritic filopodia and axons.

### Filopodia motility and stability is differentially modulated by Cdc42 (CA)-Palm, GAP 1–14, NLG-1 and Shank1b

**Figure 3:**
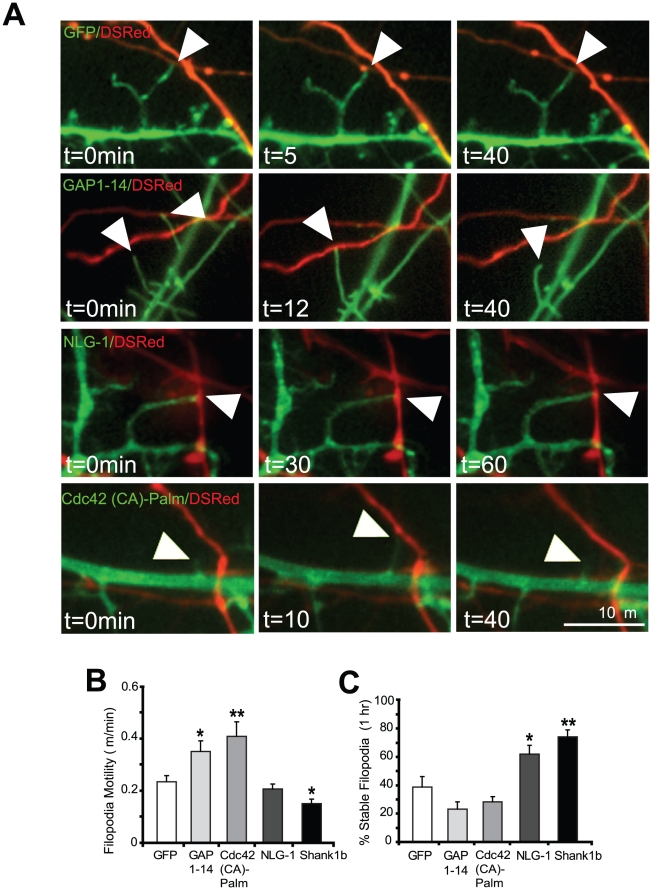
Filopodia motility and contact formation are modulated differentially by GAP 1–14 and Cdc42 (CA)-Palm versus NLG-1 and Shank1b. (A) Representative timelapse images of cells expressing GFP, GAP 1–14, NLG-1 and Cdc42 (CA)-Palm. Arrowheads point to dendritic filopodia in contact with a DsRed labeled axon. (B) Quantification of filopodia motility from neurons expressing either GFP, GAP 1–14, Cdc42 (CA)-Palm, NLG-1 or Shank1b. Filopodia in cells expressing GAP 1–14 and Cdc42 (CA)-Palm are more motile than GFP control. Filopodia expressed by NLG-1 and Shank1b are significantly less motile than filopodia expressed by GAP 1–14 and Cdc42 (CA)-Palm. (C) Quantification of percentage of stable filopodia induced by these molecules. Filopodia were imaged for 1 h. Filopodia induced by NLG-1 and Shank1b induce more stable filopodia compared to control cells expressing GFP and neurons expressing GAP 1–14 and Cdc42 (CA)-Palm. *p<0.05, **p<0.01 Data represent mean ±SEM. Scale bar, 10 µm.

**Figure 4:**
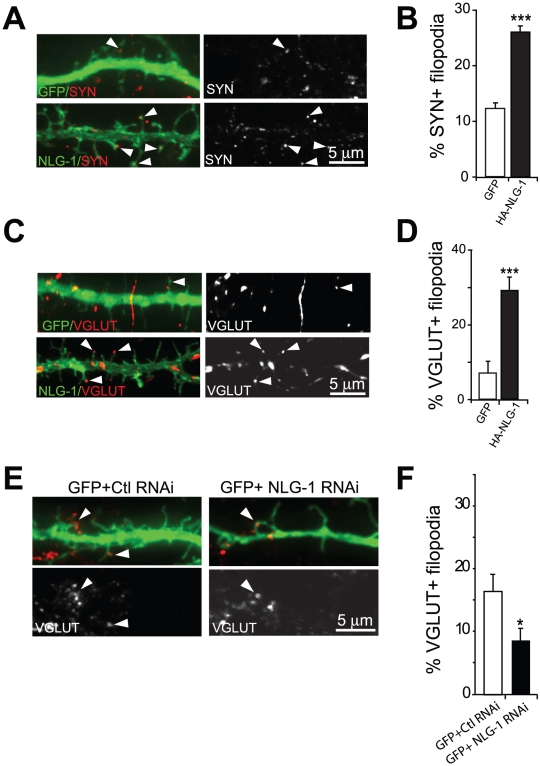
Filopodia expressing NLG-1 recruit more presynaptic clusters. (A) Expression of NLG-1 led to an increase in synaptophysin found at the tips of these filopodia compared to cells expressing GFP. Arrowheads point to dendritic filopodia in contact with a presynaptic cluster. (B) Quantification of the percentage of filopodia apposed to a cluster of synaptophysin. NLG-1 showed a two-fold increase in the percentage of synaptic filopodia compared to GFP expressing cells. (C and D) Representative images and quantification of NLG-1 led to an increase in VGLUT found at the tips of filopodia compared to cells expressing GFP. (E and F) Representative images and quantification of neurons expressing NLG-1 RNAi or Ctl RNAi with GFP. NLG-1 knockdown by RNAi led to a significant reduction in the percent of filopodia in contact with VGLUT. At least 13 cells were analyzed for each group and were collected from 3 independent experiments. *p<0.05, ***p<0.001 Data represent mean ±SEM. Scale bars, 5 µm.

To further understand what role filopodia motility and stability play in the formation of stable contacts, timelapse imaging of dually labeled neurons was performed. Contact formation was visualized between DsRed-labeled axons and cells expressing GFP-tagged GAP 1–14, Cdc42 (CA)-Palm, NLG-1 or Shank1b. Neurons expressing GAP 1–14 or Cdc42 (CA)-Palm show more transient filopodia-axon contacts over 1 h, as compared to GFP expressing cells (0.35±0.04 µm/min and 0.41±0.06 µm/min respectively, versus 0.23±0.02 µm/min for GFP; Figure 3A and B; Movie S5 and S6). In contrast, neurons expressing NLG-1 or Shank1b showed relatively less motile filopodia (0.21±0.02 µm/min and 0.15±0.01 µm/min, respectively) compared to GAP 1–14 or Cdc42 (CA)-Palm expressing filopodia (Movie S5 compared to Movie S7). This is in agreement with the finding (Figure 4) that NLG-1-expressing cells have a greater percentage of filopodia that can form synaptic contacts or ‘protosynapses’ [30], [31]. Finally, filopodia induced by NLG-1 or Shank1b were significantly more stable compared to filopodia expressed by GFP, GAP 1–14 or Cdc42 (CA)-Palm (Figure 3C). This would suggest that both filopodia motility and stabilization (following axonal contact) are necessary to induce structures that mature into synapses.

### Neuroligin-1 overexpression enhances the production of filopodia and modulates dendritic contact formation with presynaptic elements

Studies have demonstrated a role for adhesion molecules in the formation of synapses [32], [33]. Here, we wanted to investigate whether filopodia induced by NLG-1 can participate in synaptic contact formation. To answer this question, cells overexpressing NLG-1 were fixed and immunostained for endogenous synaptophysin. Our analysis revealed that a proportion of filopodia in control GFP expressing cells were positive for synaptophysin (Figure 4A and B). Moreover, NLG-1 overexpression caused an increase in the fraction of synaptophysin-positive filopodia (26.5%±1.30% compared to 11.7%±0.9% for GFP, Figure 4B), suggesting that these protrusions represent emerging synapses, or protosynapses. To characterize the type of synapses formed on filopodia, we immunolabeled GFP and NLG-1 transfected cells with the excitatory presynaptic marker VGLUT (vesicular glutamate transporter-1). We find that a fraction of VGLUT positive synapses are formed at the tips of filopodia (Figure 4C and D). Moreover, NLG-1 overexpression enhances the proportion of filopodia positive for VGLUT when compared to GFP expressing cells (29.3%±2.8% and 7.7%±2.9%; Figure 4C and 4D). Taken together, these findings are consistent with a proposed role of dendritic filopodia in excitatory synapse formation [2], [16], [27], [34], [35], [36].

We next wanted to address whether filopodia expressing NLG-1 were essential for VGLUT clustering. To address this issue we used a knockdown approach using a specific RNAi target sequence (see Materials and Methods). We found that upon expression of GFP+NLG-1 RNAi (8.6±1.8%; Figure 4E,F) there was a dramatic reduction in the percentage of filopodia contacting VGLUT clusters compared to expression of the control GFP+Ctl RNAi (16.5±2.7%; Figure 4E,F). These results demonstrate a critical role for NLG-1 in the formation of dendritic filopodia and the increase probability that these filopodia will form synaptic contacts.

### Recruitment of synaptophysin at contact sites is modulated by NLG-1

Rapid recruitment of presynaptic elements to nascent neuronal contacts is thought to be critical for synapse formation [27], [28], [35], [37]. We have previously shown that clusters of postsynaptic proteins enhance the recruitment of synaptophysin positive transport packets to contact sites [21]. Here, we examined whether dendritic filopodia associated with synaptophysin-DsRed labeled axons participate in recruiting presynaptic elements to contact sites. Our analysis reveals that 28.0%±3.6% of stable filopodia from GFP-expressing cells were found associated with synaptophysin-DsRed positive clusters, whereas 61.4%±7.9% of filopodia in NLG-1 expressing cells were associated with synaptophysin-DsRed clusters within the imaging period (Figure S3A and SB). These data are consistent with our immunostaining analysis showing that filopodia can be associated with synaptophysin positive puncta (Figure 4).

## Discussion

Dendritic filopodia have been implicated in neuronal contact formation and spine development [2], [8], [34], [35], [38], [39], [40]. It is generally assumed that in the developing neuron a filopodium is first formed; following contact with an afferent fiber, it retracts and becomes a spine [34], [41]. During development, dendritic filopodia show high motility and their numbers correlate inversely with the onset of more stable spines and synapses [2], [8], [28], [34], [42]. These observations led to the hypothesis that filopodia may initiate synaptogenesis by extending themselves towards axons and, subsequently, stabilizing the resulting connections into mature synapses [43]. This hypothesis may also be true in mature neurons. Within hours following activity blockade with tetrodotoxin (TTX), filopodia grow from existing spines, indicating that they are being used as a means of searching for glutamate-releasing presynaptic terminals [44]. Consistent with this idea, another study found that blocking synaptic transmission resulted in an increase in filopodia along dendrites as measured by electron microscopy [45]. These studies suggest that dendritic filopodia seek new presynaptic partners in order to establish new synaptic contacts.

### Increased filopodia density and motility are not necessarily correlated with synaptic contact formation

In this study we found that increased filopodia density was not correlated with synaptic contact formation. In fact, expression of Cdc42 (CA)-Palm and the palmitoylated motif GAP 1–14 led to an increase in filopodia motility, but reduced the probability of forming stable contacts with neighboring axons and the recruitment of presynaptic elements. In contrast, NLG-1 was capable of both inducing filopodia formation and transforming filopodia to spines upon contact with a presynaptic terminal.

In contrast to the extensive understanding of molecular cues controlling maturation of spines, the mechanisms and molecules involved in contact formation leading to the establishment of a synapse are far from clear. Our results are consistent with previous findings that changes in filopodia density are not necessarily correlated with synapse formation. Another hypothesis is that filopodia motility may predict the probability of initiating a stable synaptic contact. However, the evidence as to how motility correlates to synaptogenesis (ie. proportional or inversely proportional) is controversial. For example, one study showed that disrupting EphB expression decreased filopodia motility, which was correlated with a reduced rate of synaptogenesis [18]. In another study, it was found that overexpression of M6a, a neuronal glycoprotein resulted in an increase in filopodia motility and the motility significantly decreased upon synaptic contact formation [17]. In our study, we showed that expression of the adhesion molecule NLG-1 and scaffolding molecule Shank1b dramatically reduced filopodia motility and enhanced the number of stable filopodial contacts that recruit presynaptic elements. In contrast, GAP 1–14 and Cdc42 (CA)-Palm induce the most motile filopodia among all molecules in this study (Figure 3B), but the least percentage of synaptic contacts (Figure 2E). These results suggest that filopodia motility is inversely correlated with synaptic contact formation. In addition, we found only a small fraction of emerging filopodia transform to spines. Although this process normally occurs over a period of several days, expression of Shank1b can rapidly (within hours) transform filopodia to spines [5]. Our results are consistent with previous studies, which have shown that following contact with an axon, filopodia become less motile and greater stability is achieved, resulting in the formation of dendritic spines [2], [8], [46], [47].

### Implication of cell adhesion molecules in synapse formation

Despite the focused efforts of identifying cell adhesion molecules directly involved in synaptogenesis, only two adhesion molecules have been shown to induce formation of presynaptic specializations: neuroligins and synaptic cell adhesion molecule 1 (SynCAM 1) [48]. Notably, contact with these adhesion molecules induces neurons to assemble presynaptic terminals that have physiological properties virtually identical to those formed between neurons. Neuroligins are important molecules for neurodevelopment as mutations in neuroligin genes are linked to autism and mental retardation [49], [50], [51], [52], [53], [54], [55].

**Figure 5:**
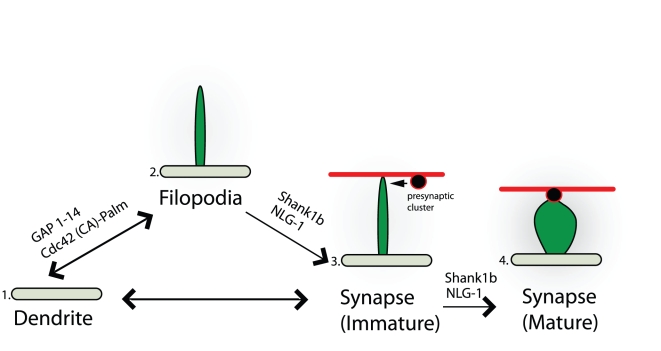
Model illustrating how filopodia induced by different molecules participate in the formation of immature and mature synapses. (1. to 2.) Molecules such as, GAP 1–14 and Cdc42 (CA)-Palm, participate in the induction of filopodia and these protrusions are mainly transient and immature. (2. to 4.) In contrast, molecules such as, NLG-1 and Shank1b, participate in the formation of more mature synapses (containing synaptic machinery such as synaptophysin and filopodia transform into a more spine-like morphological shape) possibly through the stabilization of dendritic filopodia. (1. to 4.) In addition, synapses can form independent of filopodia.

Here we show that NLG-1, a potent inducer of synapses, is also required for dendritic filopodia formation, as our knockdown data demonstrates that loss of NLG-1 causes a reduction in the percentage of synaptic contacts formed by filopodia-like protrusions (Figure 4). This suggests that one mechanism by which NLG-1-expressing filopodia could form synaptic contacts is by sampling the environment for potential axonal partners. Once contact is made these filopodia remain stable and possibly transform into dendritic spines. Interestingly, Kayser et al., (2008) observed both *in vitro* and *in vivo* that filopodia induced by EphB, a member of the receptor tyrosine kinase family, play more of an exploratory role, as they are more motile [18]. Elimination of EphB from the brain causes filopodia to become less motile and the rate of synaptogenesis decreases. This molecule behaves differently from the palmitoylation motif, GAP 1–14 and Cdc42 (CA)-Palm, as we found that motility of filopodia induced by GAP 1–14 is inversely correlated with synaptogenesis (more motility, less synaptogenesis), whereas motility of filopodia induced by EphB is proportionally correlated with synaptogenesis (less motility, less synaptogenesis) (Figure 5). In addition, expression of EphB resulted in more motile filopodia, which is opposite to the behavior of filopodia induced by NLG-1 and Shank1b. However, EphB, NLG-1 and Shank1b produce similar results, which is to increase synaptogenesis as we found that the filopodia expressed by NLG-1 and Shank1b were more stable. This suggests two things: one, that there are factors at play intrinsically related to the specificity of each protein and its role in the developing brain, two, the stability of filopodia induced by NLG-1 and Shank1b may be important for the construction of future synapses (Figure 5).

Several studies have reported that synaptic contacts can form at the tips of dendritic filopodia, resulting in filopodia stabilization and functional presynaptic boutons [2], [56]. In our study, we also observed that filopodia induced by NLG-1 were able to recruit synaptophysin-positive transport packets to sites of contact and we speculate that this is the beginning of a protospine, which may later develop into a functional dendritic spine (Figure 5). Together, these findings provide a novel mechanism by which NLG-1 could form dendritic spines by promoting filopodia extension and stabilizing contact with a presynaptic terminal. This is followed by stabilization of the contact resulting in filopodia retraction and further spine development. Thus we support data showing that NLG-1 is a key molecule for spine formation during development.

### Implication of scaffolding molecules in synapse formation

Previous work suggests that scaffolding proteins may help stabilize filopodia to form dendritic branches. In Zebrafish tectal neurons, timelapse imaging showed when a filopodium bearing PSD-95 puncta undergoes retraction, distal regions retract normally, but retraction is halted when a PSD-95 punctum is encountered [36], [57]. Thus, PSD-95 accretion strongly correlates with the stabilization of a filopodium and its maturation into a dendritic branch. Similarly, work done by Prange et al., 2001 found using timelapse imaging of cultured cortical neurons that filopodia containing PSD-95 clusters were significantly more stable than those lacking clusters and led to an increase in the number of synapses formed [58].

Similarly, we found that filopodia containing clusters of Shank1b were less dynamic and led to an increase in the number of spines formed [5], suggesting that these filopodia function to make stable contacts, consequently leading to the formation of a synapse. Similar to PSD-95, it is possible that Shank1b containing clusters are also trafficked to filopodia in a developmentally regulated manner and this is associated with increased filopodia stability and synapse formation.

Unlike NLG-1, which interacts with its presynaptic counterpart neurexin, to enhance the number of synapses, Shank1b likely induces spinogenesis through the stabilization of the cytoskeleton. These findings raise the question how does Shank1b communicate with presynaptic sites to enhance synaptic contact formation? It has been previously shown that transport of synaptophysin to sites opposed to stationary clusters of PSD-95 caused rapid morphological rearrangements of the newly recruited clusters [21]. This finding suggests that postsynaptic scaffolds can recruit axonal transport packets for initiation and/or stabilization of new sites of contact [21]. Therefore, it is possible that expression of Shank1b may trigger recruitment and morphological changes of presynaptic complexes and this process may be critical for stabilization of dendritic filopodia.

### Possible limitations of this study and future directions

Although we provide evidence that filopodia induced by specific proteins can participate in contact and synapse formation, there are three key limitations to this study that will be addressed here. First, the consequences of photodamage on cellular viability can be severe [59], [60] and some studies have reported that sampling the specimen for long durations increases the probability that the neuron will show abnormal physiological processes [59], [60]. Thus, we are aware that we may have ‘missed’ events whereby the fate of the filopodium was continually changing in these non-imaged time periods. However, fewer sampling time points were purposefully selected to ensure cell viability. Second, we used the palmitoylation motif GAP 1–14, as a molecular tool, to examine factors that stimulate synaptogenesis in the developing brain. Our findings regarding the palmitoylation motif GAP 1–14 in dendritic filopodia induction and synaptogenesis are not representative of the endogenous function of GAP-43. As a matter of fact, in presynaptic axons, phosphorylation of GAP-43 by PKC in growth cones and nascent synapses is required for synaptogenesis [61]. Clearly, since GAP 1–14 is lacking the phosphorylation motif, it is not being phosphorylated. Therefore, the use of the GAP 1–14 motif in this study is not to conclude any biological or functional property of this molecule. Rather, we use it as an excellent molecular tool to manipulate filopodia outgrowth and stability in order to evaluate its role in contact formation. Third, it is important to mention that different types of filopodia serve distinct functions in the brain. For example, Portera-Cailliau et al., 2003 demonstrated that axonal filopodia are important for searching for appropriate postsynaptic partners [15]. Conversely, dendritic filopodia may be important for the formation of dendritic spines. And finally, there are filopodia that serve as precursors for dendritic branching. Different filopodia serve different functions and may require distinct molecular machinery for their roles. For the purpose of this study, we only focused on the role of filopodia as precursors of spines for synaptogenesis.

In the future, it will be important to examine the function of spine synapses induced by the expression of NLG-1 alone, and to determine whether overexpression of its binding partner neurexin is also required. A recent paper suggests that the NLG-1-neurexin interaction may be critical for filopodia stability and synapse formation [31]. In addition, it would be interesting to examine filopodia dynamics in cultured hippocampal neurons taken from transgenic animals overexpressing NLG-1. This experiment would be a further test of our hypothesis that filopodia expressing NLG-1 are more likely to form synaptic contacts leading to filopodia stability and possible spine formation.

## Materials and Methods

### Ethics Statement

Protocols were approved by the Animal Care Committee consistent with Canadian Council on Animal Care and Use Guidelines (University of British Columbia, Animal Care Committee, Neuroplasticity, A09-0665).

### cDNA cloning, RNAi and construction

GAP 1–14 and Cdc42 (CA)-Palm plasmids were constructed as previously described by [5], [23]. And GFP tagged Shank1b, HA and GFP tagged NLG-1 were constructed as previously described by [19], [62], [63]. NLG-1 RNAi sequence was used as previously described [64] and re-cloned into the pSUPER vector. Previously used NLG-1 forward primer GATCCCCTGGAAGGTACTGGAAATCTATTCAAGAGATAGATTTCCAGTACCTTCCTTTTTTCA and the reverse primer used AGCTTGAAAAAAGGAAGGTACTGGAAATCTATCTCTTGAATAGATTTCCAGTACCTTCCAGGG (Dharmacon Inc.). The restriction sites used in the pSUPER vector were BglII and HindIII. This sequence was transfected into rat hippocampal neurons to suppress expression of endogenous NLG-1.

### Hippocampal Cultures and Cell Transfection Methods

Hippocampal neurons were prepared from embryonic day 18/19 rat pups as previously described [5], [21]. For experiments involving fixed cells, immediately after dissection and digestion, neurons were plated at a density of 150,000 cells/well of a 24 well plate. For cell transfection, we used Lipofectamine 2000 (Invitrogen). Briefly, we used 1–1.5 µg/µL of DNA and 0.8 µL of lipofectamine 2000 per well and incubated for 2–3 hrs at which time the Neural Basal Media (NBM) was removed and replaced with original NBM. For live cell imaging experiments, hippocampal cultures were transfected by nucleofection (Amaxa), by lipid-mediated gene transfer (Invitrogen), or using a calcium phosphate transfection kit (BD Biosciences, CA). Similar results were obtained with each protocol. Briefly, the electroporation protocol is as follows: 6 million cells were re-suspended in 100 µl of room temperature electroporation solution (120 mM KCl, 10 mM KH_2_PO_4_, 0.15 mM CaCl_2_, 5 mM MgCl_2_, 25 mM HEPES, 2 mM EGTA, 2 mM ATP, 5 mM GSSG, pH to 7.4) with 2 µg of high quality endotoxin-free DNA. Neurons were then transfected by electroporation, as described by AMAXA Inc Amaxa (Gaithersburg, MD). Cells were plated at a final density of 0.5 million/mL and allowed to recover in DMEM with 10% Calf Serum for 1 hour before replacement with NBM (Invitrogen). Calcium phosphate transfections were done at 7 days in vitro [54]: briefly, 2 µg of DNA and 6.2 µl of calcium phosphate buffer (4 M, BD Biosciences) were mixed with 92 µl of HBSS (Hanks balanced salt solution, pH 7.0) and let stand for 5 minutes at room temperature. This DNA solution was added drop-wise to 100 µl of distilled water and the mix was added to the cells with 500 µl of NBM per well. Cells were incubated for 10 minutes at 37°C and the calcium phosphate reagent was replaced with original NBM.

### Fixation and Immunocytochemistry

Hippocampal neurons were fixed with 2% PFA and 4% sucrose or with methanol at −20°C when staining for synaptic proteins. Cells were then washed three times with phosphate buffer saline (PBS) containing 0.3% triton to permeabilize cells. The following primary antibodies were used: GFP (chicken; 1∶1000;AbCam), HA (mouse; 1∶1000; Synaptic Systems), Synaptophysin (1∶1000; Zymed), MAP-2 (1∶1000; Pharmingen), GluR1 (rabbit; 1∶500; Upstate Biotech) and PSD-95 (1∶1000; ABR). We used the following secondary antibodies: Alexa 488-conjugated anti-chicken (1∶1000; Molecular Probes), Alexa 568-conjugated anti-mouse (1∶1000; Molecular Probes) and Alexa 568-conjugated anti-rabbit (1∶1000; Molecular Probes). Coverslips were incubated for 1 h at room temperature with primary and secondary antibodies.

### Microscopy and Timelapse Imaging

For all experiments, images were collected on a Zeiss Axiovert M200 inverted light microscope. Images were taken using a 63×1.4 NA oil immersion objective and a monochrome 14-bit Zeiss Axiocam HR charged-coupled camera. To minimize potentially out of focus images, z stacks were collected (0.5 µm increments) and projected into a single image. For timelapse imaging experiments, a single plane of focus was used to capture movies (1 frame/min) and this was done to minimize photobleaching and toxicity. For these experiments, to decrease the possibility of out-of-focus protrusions, we manually monitored the focus of live cells. Cells were imaged at 37°C in a sealed incubation chamber, supplemented with 5% CO_2._

### Quantitative measurement of filopodia and dendritic spines

All protrusions were measured on all dendrites within the field of view and an observer blinded to the transfection type did all analyses. Protrusions were scored based on their morphology. Protrusions that ranged from 1–10 µm without a visible head were counted as filopodia and protrusions with a bulbous head wider than its base were counted as spines [5], [39]. Spines had to have a head size of 0.5 µm or greater to be counted as a spine. Analyses were performed using Northern Eclipse Software (Empix Imaging Inc.). All statistical analysis was done using XLSTAT add-in for Microsoft Excel (Addinsoft, NY) or student's T-test (Microsoft Excel) and multiple group comparisons were done using the one-way analysis of variance (ANOVA, with Student-Newman-Keuls post-hoc correction).

### Calculation of synaptophysin cluster mobility

Movement of synaptophysin-positive clusters was analyzed using Image J (Wayne Rasband, NIH). Images were corrected for drift (RegisterROI, Michael Abramoff, University of Iowa Hospitals and Clinics, USA), and velocities were recorded (Manual Tracker, Fabrice Cordelières, Institut Curie, France). Discrete puncta of synaptophysin fluorescence were classified as “clusters” if they were at least 1.5 times greater than the average intensity of the background axon. Synaptophysin clusters were scored as “stable clusters” if they did not move more than 2 µm over the entire image acquisition period or “splitting” if a single cluster split into 2 separate clusters. All other clusters were classified as “moving clusters”. Changes in position that were less than 0.2 µm (2 pixels for non-binned images) per time point were omitted.

### Calculation of synapse number and size

Images were exported as 16 bit and analyzed using Northern Eclipse software as previously described [5]. Briefly, images were processed at a constant threshold level to create a binary ‘mask’ image, which was multiplied by the original image. The resulting image contained a discrete number of clusters with pixel values of the original image. Only clusters with average pixel intensity 1.5 times greater than background pixel intensity were used for analysis. In addition, only dendritic processes were used for analyses (cell bodies and axons were excluded). The density of PSD-95 puncta is expressed per area of dendrite (µm^2^) and normalized to GFP-expressing neurons.

## Supporting Information

Figure S1 **A small percentage of filopodia can transform into spines and this process requires several days.** (A) A representative image of a whole neuron expressing GAP 1–14 on DIV 10, 11 and 12 which has been retro-immunolabeled for GluR1. Lower images (containing a boxed region) show a filopodia on DIV 10 that later becomes a spine and contains a GluR1 puncta on DIV 12. (B) Filopodia expressing either GFP or GAP 1-14-GFP were imaged once per day for 3 days to determine their fate. (C) Quantification of spines that formed independently of filopodia. Approximately 30% of spines from neurons expressing either GFP or GAP 1-14-GFP emerged de novo. Scale bar, 10 µm.
(TIFF)

Figure S2 **A role for dendritic filopodia in exploration and synaptic contact formation.** (A) Electroporation of a DsRed construct was used to label axons of one cell and GFP was used to fill a different cell. Images were captured every 1 min for 1 h total. (B) Quantification of filopodia revealed that filopodia appeared to continuously interact with axons en passant. A small percentage of filopodia formed new and stable contacts throughout the imaging period. ***p<0.001 Data represent mean ±SEM. Scale bar, 5 µm.
(EPS)

Figure S3 **Recruitment of synaptophysin to sites containing NLG-1 induced filopodia.** (A) Representative timelapse images of cells expressing Synaptophysin-DsRed and either GFP or NLG-1. Arrowheads indicate filopodia in contact with clusters of synaptophysin. Arrows denote filopodia in contact with axons labeled with Synaptophysin-DsRed, but do not contain a synaptic cluster. (B) Cells expressing NLG-1 showed a dramatic increase in the percent of filopodia contacting presynaptic clusters compared to control cells expressing GFP. *p<0.05, **p<0.01, ***p<0.001 Data represent mean ±SEM. Scale bars, 5 µm.
(EPS)

Movie S1 **Transient contacts between dendritic filopodia and axon.** Timelapse imaging of GFP-expressing dendritic filopodia formed transient contacts with a DsRed labeled axon (one image was acquired every min).
(MOV)

Movie S2 **Filopodia form new contacts in neuronal cells.** Timelapse imaging of GFP-expressing dendritic filopodia formed new contacts with a DsRed labeled axon (one image was acquired every min).
(MOV)

Movie S3 **Filopodia form stable contacts in neuronal cells.** Timelapse imaging of GFP-expressing dendritic filopodia formed stable contacts with a DsRed labeled axon (one image was acquired every min).
(MOV)

Movie S4 **Axonal growth cone contacts dendrite.** Images were acquired every 1 min for a period of 1 h. A DsRed labeled axonal growth cone initiated contact with a dendrite and stimulated the growth of a dendritic filopodia.
(MOV)

Movie S5 **Filopodial dynamics in neuronal cells expressing GAP 1–14.** Cultured hippocampal neurons transfected with the palmitoylation motif of GAP-43 (GAP 1–14) and imaged for 1 hr (one image every 1 min) showed dynamic filopodia-like protrusions at DIV 8.
(MOV)

Movie S6 **Filopodial dynamics in neuronal cells expressing GFP.** Cultured hippocampal neurons transfected with GFP and imaged for 1 h (one image every 1 min).
(MOV)

Movie S7 **Filopodial dynamics in neuronal cells expressing NLG-1.** Cultured hippocampal neurons transfected with NLG-1 and imaged for 1 hr (one image every 1 min) revealed mostly stable filopodia.
(MOV)
