# Supplementary figures and images for: Proteins That Promote Filopodia Stability, but Not Number, Lead to More Axonal-Dendritic Contacts
Source: PLoS One. 2011 Mar 7;6(3):e16998. doi: 10.1371/journal.pone.0016998 (PMC3049770; doi:10.1371/journal.pone.0016998)

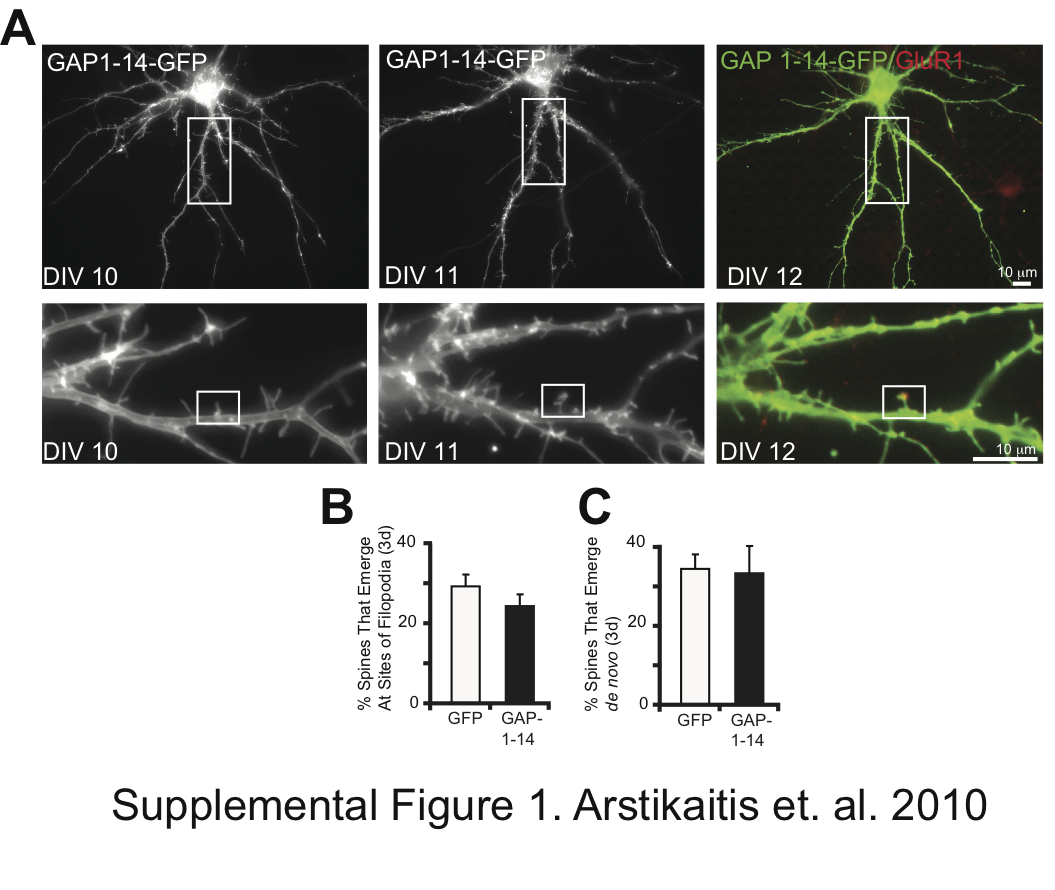

Supplement: Figure S1 — A small percentage of filopodia can transform into spines and this process requires several days. (A) A representative image of a whole neuron expressing GAP 1–14 on DIV 10, 11 and 12 which has been retro-immunolabeled for GluR1. Lower images (containing a boxed region) show a filopodia on DIV 10 that later becomes a spine and contains a GluR1 puncta on DIV 12. (B) Filopodia expressing either GFP or GAP 1-14-GFP were imaged once per day for 3 days to determine their fate. (C) Quantification of spines that formed independently of filopodia. Approximately 30% of spines from neurons expressing either GFP or GAP 1-14-GFP emerged de novo. Scale bar, 10 µm. (TIFF) [file pone.0016998.s001.tiff]
